# Structural Properties of Graphene Oxide Prepared from Graphite by Three Different Methods and the Effect on Removal of Cr(VI) from Aqueous Solution

**DOI:** 10.3390/nano13020279

**Published:** 2023-01-09

**Authors:** Feng Gao, Lei Zhang, Libin Yang, Xuefei Zhou, Yalei Zhang

**Affiliations:** 1 State Key Laboratory of Pollution Control and Resources Reuse, College of Environmental Science and Engineering, Tongji University, Shanghai 200092, China; 2 Key Laboratory of Yangtze Water Environment for Ministry of Education, College of Environmental Science and Engineering, Tongji University, Shanghai 200092, China; 3 Shanghai Institute of Pollution Control and Ecological Security, Tongji University, Shanghai 200092, China

**Keywords:** adsorption, hexavalent chromium, graphene oxide, adsorption equilibrium, adsorption kinetics, thermodynamics

## Abstract

Herein, three types of graphene oxides (GOs, GO-M1, GO-M2 and GO-M3) have been successfully prepared from graphite by three different methods and utilized for the removal of Cr(VI) from aqueous solutions. Further, the effects of initial concentration and pH, adsorbent dosage, contact time and temperature on the adsorption performance of GOs were investigated by batch adsorption experiments. Furthermore, the adsorption mechanisms for Cr(VI) adsorption by GOs are mainly the redox reaction and electrostatic attraction, while there are also pore filling, ion exchange and complexation involved in these adsorption processes. The adsorption kinetic and isotherm data indicate that these adsorption processes of GOs on Cr(VI) are dominantly monolayer chemisorption and equilibrium can be reached in 30 min. The saturation adsorption capacities (Q_m_, 298.15 K) of GO-M1, GO-M2 and GO-M3 for Cr(VI) are estimated to be 3.5412 mg⋅g^−1^, 2.3631 mg⋅g^−1^ and 7.0358 mg⋅g^−1^, respectively. Moreover, the adsorption thermodynamic study showed that these adsorption processes of Cr(VI) by the three types of GOs at 298.15 K to 323.15 K are endothermic, entropy-driven and thermodynamically spontaneous and feasible. Overall, these findings provided vital insights into the mechanism and application of Cr(VI) removal by GOs.

## 1. Introduction

With the rapid development of the economy, the standard of living has been considerably improved, and therefore the concern for environmental issues has become increasingly strong [1,2]. Industrial wastewater as one of the three industrial wastes (solid waste, wastewater and waste gas) and how to treat it efficiently and stably at a low price remains a challenge for scientific researchers. As a heavy metal element, chromium (Cr) is commonly found in large quantities as trivalent and hexavalent [Cr(VI)] compounds in the wastewater of many industries, such as electroplating, tannery, chemical, pigment, metallurgy and so on [3,4]. All chromium compounds are toxic, notably Cr(VI) is far more toxic than chromium metal, trivalent or tetravalent chromium [5]. Cr(VI) can easily be absorbed into the body through water, air and food and is not readily biodegradable, thus accumulating in the body and resulting in serious illness [6]. Meanwhile, since Cr(VI) exists in the form of metal anions, it is weakly adsorbed by soil solutions, making Cr(VI) highly migratory in the natural environment and a great threat to the environment. Considering this, the U.S. Environmental Protection Agency identified Cr(VI) as one of the highly hazardous toxic substances [7], and China also listed it as the first category of pollutant in the integrated wastewater discharge standard (GB 8978-1996) and stipulated that its maximum allowable discharge concentration is 0.5 mg/L. In particular, China has made more stringent requirements in the discharge standard for pollutants from electroplating (GB 21900-2008), stipulating that the content of Cr(VI) shall not exceed 0.2 mg/L. Therefore, the pollution control of Cr(VI)-containing wastewater has become imminent [8].

Hitherto, numerous technologies including coagulation [9], adsorption separation [10], membrane filtration [11], ion exchange [12], electrochemical treatment [13], photocatalytic reduction [14] and microbe-based technologies [15] have been adopted for the Cr(VI) removal of Cr(VI)-contaminated water and wastewater [16]. Of these, the adsorption approach has been receiving more and more attention from researchers because of its low cost, availability, simplicity of operation, high removal efficiency, profitability and versatility in terms of adsorbent performance [17,18,19]. Graphene is a two-dimensional carbon material composed of a layer of carbon atoms packed periodically and closely in a hexagonal honeycomb structure, which enjoys high specific surface area, good chemical properties, good stability performance and mechanical property in the environment and is now widely utilized in energy storage, adsorption separation, catalysis and other fields [20,21]. Previous studies have shown calcined bauxite, zeolite and bentonite clay, etc., have been used for Cr(VI) removal from aqueous media. However, none of the adsorbents exhibited significant Cr(VI) removal efficiency and excellent adsorption capacity [22]. Graphene oxide (GO) is an oxide of graphene, which, after oxidation, exhibits a significant increase in oxygen-containing functional groups and more defective structures, thus giving it a better performance in the removal of contaminants (i.e., Cr(VI)) in aqueous media than graphene and other adsorbents [23,24]. Graphite oxide was first prepared as early as 1859 by Brodie, an Oxford chemist who treated graphite with a mixture of potassium chlorate and concentrated nitric acid, but at the time, no attention was paid to this new material [25,26]. With the development of graphene oxide, methods for its synthesis have sprung up [27]. Among them, William S. Hummers Jr.’s method (M1), Daniela C. Marcano’s method (M2) and Nina I. Kovtyukhova’s method (M3) are three representative preparation methods.

In view of this, three types of GOs (GO-M1, GO-M2 and GO-M3) were prepared by three different methods (M1, M2 and M3) and applied to the removal of Cr(VI) from aqueous solutions. Advanced characterization technologies are used to verify whether the three types of GOs are successfully prepared on the one hand and to reveal the potential mechanism of the Cr (VI) adsorption of the three types of GOs in aqueous solution on the other hand. Furthermore, various experimental parameters affecting the adsorption of Cr(VI), including the initial concentration and pH, adsorbent dosage, contact time and temperature were also investigated. Moreover, the adsorption kinetics, isotherms and thermodynamic investigations were carried out to further unravel the adsorption processes of three types of GOs on Cr(VI).

## 2. Materials and Methods

### 2.1. Materials

Graphite powder (99.95%, metals basis) was supplied by Shanghai Aladdin Biochemical Technology Co., Ltd. (Shanghai, China). Superior grade potassium dichromate and analytical grade potassium permanganate, sodium nitrate, hydrogen peroxide, sulfuric acid, phosphoric acid, hydrochloric acid, potassium persulfate and phosphorus pentoxide were purchased from Sinopharm Chemical Reagent Co., Ltd. (Shanghai, China). Deionized water was provided by the Hongkou Baoxing deionized water factory (Shanghai, China). The different concentrations of Cr(VI) solutions used for adsorption experiments were obtained by diluting the Cr(VI) standard stock solution.

### 2.2. Fabrication of GOs

**Figure 1:**
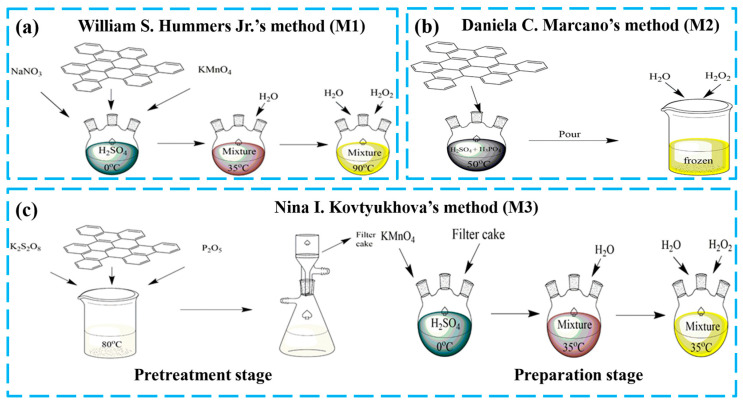
Schematic diagram of the preparation process of three types of GOs (**a**) WIilliam S. Hummers Jr.’s method, (**b**) Daniela C. Marcano’s method and (**c**) Nina I. Kovtyukhova’s method.

The three kinds of GOs utilized in this experiment were synthesized according to William S. Hummers Jr.’s method (M1) [28], Daniela C. Marcano’s method (M2) [29] and Nina I. Kovtyukhova’s method (M3) [30] and were called GO-M1, GO-M2 and GO-M3, respectively. The preparation procedure is briefly depicted in Figure 1, with more details provided in Text S1.

### 2.3. Characterization of GOs

Scanning electron microscopy (SEM) images (surface morphology of materials) of GOs and graphite and elemental mapping images (surface element distributions of materials) of GOs were determined on a field emission scanning electron microscope (S-4800, Hitachi, Tokyo, Japan) fitted with an energy dispersive spectroscopy (EDS). Raman spectra (RS) of GOs and graphite were obtained on a micro-Raman spectrometer (Invia, Renishaw, Wotton-under-Edge, UK). The thermogravimetric (TG) curves of GOs and graphite and differential scanning calorimetry (DSC) curves of GOs were determined by a comprehensive thermogravimetric analyzer (STA 409PC, Netzsch, Selb, Germany). X-ray diffraction (XRD) patterns (crystal structure of materials) of GOs and graphite were obtained by X-ray diffractometer (D8, Bruker, Massachusetts, Germany). Moreover, nitrogen adsorption and desorption isotherms and pore size distributions of GOs were measured by a specific surface area and pore size analyzer (TriStar 3000, Micromeritics, Norcross, GA, USA) and the BET-specific surface areas of GOs were calculated according to the BET method. Fourier transform infrared spectra (FTIR, functional groups on the surface of materials) of GOs were recorded by an FTIR spectrometer (Nexus 670 Nicolet, Thermo, Waltham, MA, USA). The C1s spectra of GOs before and after adsorption of Cr(VI) and the Cr2p spectra of GO-M3 after the adsorption of Cr(VI) were determined by X-ray photoelectron spectroscopy (XPS, PHI5300, Perkin Elmer, Waltham, MA, USA).

### 2.4. Adsorption Experiments

Cr(VI) adsorption experiments were carried out in conical flasks containing 20 mL of Cr(VI) solution and 15 mg of adsorbent (GO). Various experimental parameters affecting Cr(VI) adsorption were investigated, including the initial concentration of Cr(VI) (1.0~3.0 mg/L), adsorbent (GOs) dosage (5~35 mg), initial pH of Cr(VI) solution (1~11), contact time (5~35/40 min) and temperature (298.15~323.15 K). In the adsorption experiments, the pH of the Cr(VI) solution was adjusted and maintained by using HCl (1 wt%) or NaOH (1 wt%) solutions. All adsorption experiments were performed on a water bath thermostatic oscillator (SHZ-B, Shanghai Boxun Medical Biological Instrument Corp., Shanghai, China) with sufficient stirring at a fixed speed to reach adsorption equilibrium. Then, GO was separated from the solution by filtration at the end of the adsorption experiments and the residual Cr(VI) concentration in the solution was determined at 540 nm by a UV–Vis spectrophotometer (UV756CRT, Shanghai Yoke Instrument Co., Ltd., Shanghai, China) using diphenylcarbonyldihydrazide spectrophotometry [31]. The adsorption capacity at a certain time (*Q_t_*, mg/g) and at equilibrium time (*Q_e_*, mg/g), and removal rate (*R*, %) were calculated according to Equation (1) [32], Equation (2) [33] and Equation (3) [34]:

(1) $$Q_{t}=\frac{\left(C_{0}-C_{t}\right)\times V}{m}$$

(2) $$Q_{e}=\frac{\left(C_{0}-C_{e}\right)\times V}{m}$$

(3) $$R=\frac{C_{0}-C_{e}}{C_{0}}\times 100\%$$

where *C_0_* (mg/L), *C_t_* (mg/L) and *C_e_* (mg/L) refer to the initial, time t and equilibrium concentrations of the Cr(VI) solution, respectively; *m* (g) denotes the dry weight of GO; *V* (L) represents the volume of the Cr(VI) solution.

### 2.5. Adsorption Kinetics

Adsorption kinetics investigations are essential to determine the effectiveness of the adsorption process as they provide much valuable information, such as adsorption rates, adsorbent properties and mass transfer mechanisms, among others [35]. Here, nonlinear pseudo-first-order kinetics (Equation (4)) and pseudo-second-order kinetic (Equation (5)) models [36] were exploited to fit the adsorption kinetic data for Cr(VI) adsorbed by GOs.

(4) $$Q_{t}=Q_{e}\left(1-e^{-k_{1}t}\right)$$

(5) $$Q_{t}=\frac{Q_{e}^{2}k_{2}t}{1+Q_{e}k_{2}t}$$

where *Q_t_* (mg/g) and *Q_e_* (mg/g) are the adsorption capacity at time *t* (min) and at equilibrium, respectively; *k_1_* (min^−1^) and *k_2_* (g⋅mg^−1^⋅min^−1^) are the rate constants of the pseudo-first-order and pseudo-second-order models, respectively.

### 2.6. Adsorption Isotherms

At constant temperatures, adsorption isotherm models may depict the interaction processes between the adsorbent and the adsorbate. A clear understanding and interpretation of adsorption behavior at equilibrium is therefore a critical step in predicting the adsorption processes of diverse adsorption systems [37]. Here, linear Langmuir (Equation (6)) [38] and Freundlich (Equation (7)) [39] isotherm models were applied to fit the adsorption isotherm data for Cr(VI) adsorbed by GOs.

(6) $$\frac{C_{e}}{Q_{e}}=\frac{1}{Q_{m}}C_{e}+\frac{1}{Q_{m}K_{L}}$$

(7) $$\ln Q_{e}=\frac{1}{n}\ln C_{e}+\ln K_{F}$$

where *Q_e_* (mg/g) and *Q_m_* (mg/g) represent the adsorption capacity at equilibrium and at maximum, respectively; *K_L_* (L⋅mg^−1^) and *K_F_* ((mg⋅g^−1^)⋅(L⋅mg^−1^)^1/n^) are Langmuir and Freundlich isotherm constants, respectively; and *C_e_* (mg/L) is the equilibrium concentration of the Cr(VI), *n* stands for adsorption intensity.

### 2.7. Adsorption Thermodynamics

To better understand the feasibility and mechanism of adsorption, three thermodynamic parameters, including Gibbs free energy change of adsorption (Δ*G^θ^*, (kJ⋅mol^−1^)), adsorption enthalpy change (Δ*H^θ^*, (kJ⋅mol^−1^)) and entropy change of adsorption (Δ*S^θ^*, (J⋅mol^−1^⋅k^−1^)) were accurately calculated to obtain in-depth information about the adsorption of CR on GOs.

More specifically, Δ*G^θ^* can be calculated from Equation (8) [40].

(8) $$\Delta G^{\theta }=-RT\ln K_{d}=-RT\ln\frac{Q_{e}}{C_{e}}$$

In addition, the relationship of Δ*G^θ^* to Δ*H^θ^* and Δ*S^θ^* can be described by Equation (9) [41].

(9) $$\Delta G^{\theta }=\Delta H^{\theta }-T\Delta S^{\theta }$$

Moreover, substituting Equation (8) into Equation (9) and undergoing a simple transformation can give Equation (10) [42]:

(10) $$\ln\frac{Q_{e}}{C_{e}}=-\frac{\Delta H^{\theta }}{RT}+\frac{\Delta S^{\theta }}{R}$$

where *K_d_* is the distribution constant, *Q_e_* (mg/g) represents the adsorption capacity at equilibrium, *C_e_* (mg/L) is the equilibrium concentration of the Cr(VI), *R* stands for gas constant (8.314 J·mol^−1^·K^−1^) and *T* (K) is the absolute temperature.

Finally, based on the linear relationship between ln(*Q_e_*/*C_e_*) and 1/*T*, we can draw a straight line and the values of Δ*H^θ^* and Δ*S^θ^* can be obtained from the slope and intercept, respectively.

## 3. Results and Discussions

### 3.1. Characterization of GOs

The surface morphology, degree of defects, crystal structure and thermal stability of graphite powder and three types of GOs (GO-M1, GO-M2 and GO-M3) were determined by SEM, RS, TG and XRD, respectively. In addition, the surface elements, thermal effects, pore properties and surface functional groups of the three types of GOs were characterized by EDS, DSC, BET and FTIR, respectively. Moreover, the changes of three types of GOs before and after Cr(VI) adsorption were characterized by XPS.

#### 3.1.1. SEM-EDS Analysis

**Figure 2:**
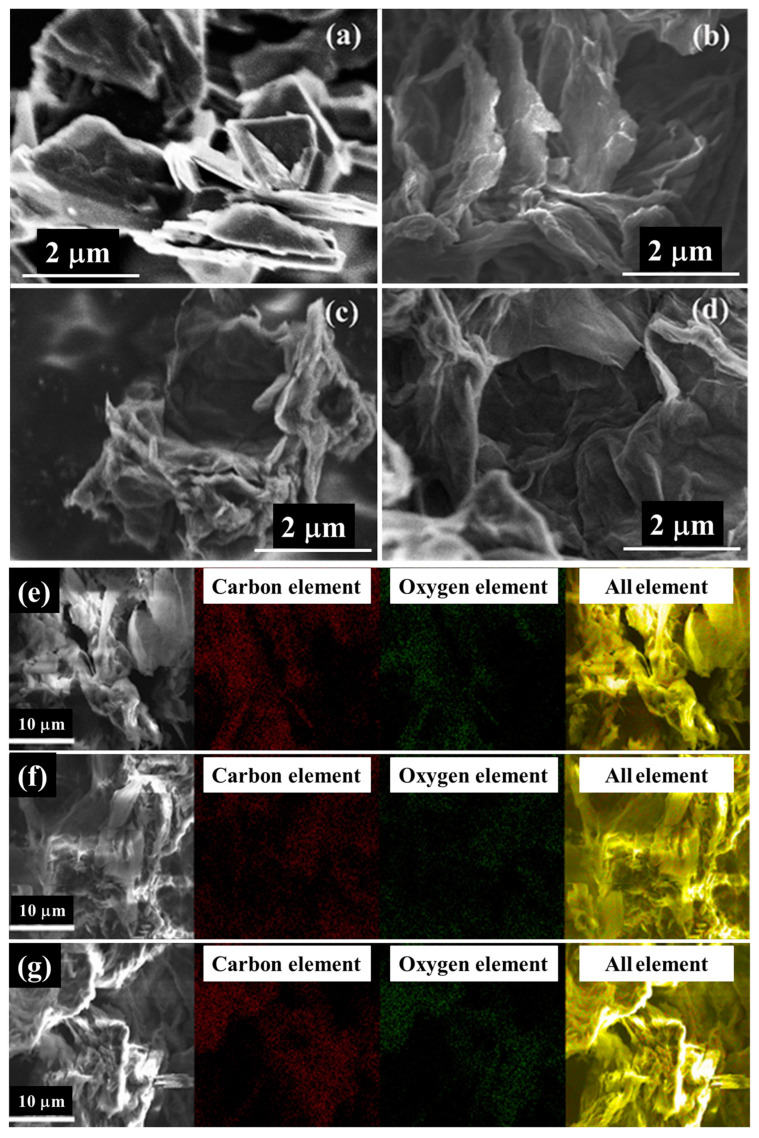
SEM images of graphite powder and three types of GOs: (**a**) graphite powder, (**b**) GO-M1, (**c**) GO-M2 and (**d**) GO-M3. SEM images and their corresponding elemental mapping images of three types of GOs: (**e**) GO-M1, (**f**) GO-M2 and (**g**) GO-M3.

The SEM images of graphite powder shows that the graphite powder exhibits a flat surface and a regular thicker sheet-like morphology (Figure 2a). However, these GOs give the appearance of thin and randomly aggregated nanosheets tightly packed together and normally with a wrinkled sheet-layer morphology (Figure 2b–d). This indicates that the ordered lamellar morphology in the original graphite was destroyed due to oxidation, showing a disordered lamellar morphology [25]. In addition to this, GOs possess darker SEM images relative to graphite powder, which can be attributed to the poor electrical conductivity of GOs [43].

The mapping images (Figure 2e–g) and EDS element semi-quantitative analysis results (Table 1) revealed that all three types of GOs are dominated by carbon and oxygen elements and the content of impurity elements is extremely low, which indicates that the samples were thoroughly cleaned during the preparation process and the samples were relatively pure. Furthermore, the high presence of oxygen elements in these GOs indicates that all three methods (M1, M2 and M3) were able to successfully prepare GOs. The percent oxygen atoms of GO-M1, GO-M2 and GO-M3 are 30.70%, 33.38% and 35.68%, respectively, and the oxygen contents of these GOs are relatively close, with GO-M3 containing slightly more oxygen than the other two types of GOs.

#### 3.1.2. RS Analysis

**Figure 3:**
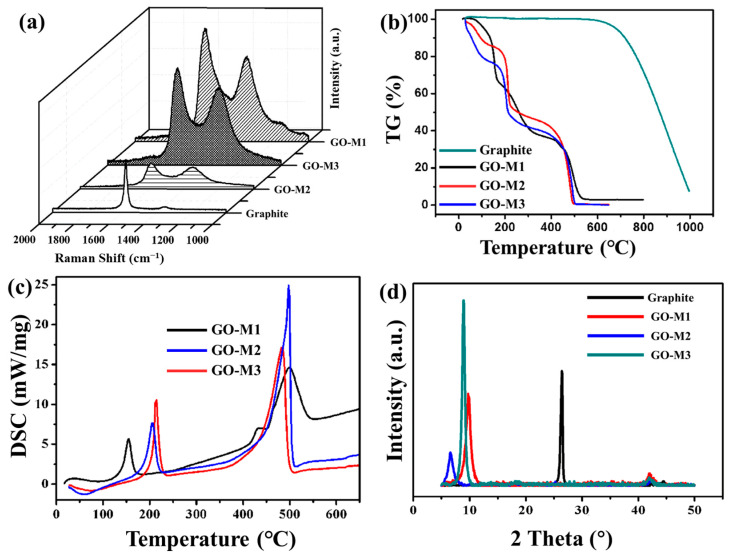
RS patterns (**a**), TG curves (**b**) and XRD patterns (**d**) of graphite powder and three types of GOs and DSC curves of GOs (**c**).

The RS of graphite powder and three types of GOs have been provided in Figure 3a. In all RS, two distinct peaks were found, the D peak at 1352 cm^−1^ and the G peak at 1578 cm^−1^. It is known that the D-band is associated with structural defects, amorphous carbon or edges that may break the symmetry and selection rules, which are not seen in highly crystalline graphite/graphene samples; the G-band corresponds to the in-plane vibration of the graphite structure [44]. As the damage to the molecular skeleton of graphite deepens, the D-peak increases and the G-peak gradually decreases. Therefore, in practice, the intensity ratio of D-peak to G-peak (I_D_/I_G_) is widely used to characterize the degree of defects in graphite [45]. Table 2 lists the I_D_/I_G_ values of graphite powder and three types of GOs. The I_D_/I_G_ values of GOs are significantly larger than those of graphite, which indicates that graphite possesses more defective structures after being oxidized; the I_D_/I_G_ value of GO-M3 is the largest among the three types of GOs, which demonstrates that M3 causes deeper oxidation to graphite powder and damages the graphite molecular structure the most.

#### 3.1.3. TG-DSC Analysis

TG curves of graphite and GOs and DSC curves of GOs are shown in Figure 3b,c. As presented in Figure 3b, the mass losses of 4%, 10% and 20% for GO-M1, GO-M2 and GO-M3, respectively, before 100 °C can be attributed to the evaporation of water molecules enclosed between the GO sheets or adsorbed by the GOs [46]; the mass losses of GO-M1, GO-M2 and GO-M3 at the stage around 100–200 °C were 30%, 20% and 30%, respectively, which can be attributed to the pyrolysis of oxygen-containing functional groups [47]. These GOs also showed a large mass loss around 500 °C, which might indicate that the remaining destroyed carbon skeleton was heavily thermally degraded at this stage. Compared with the TG curves of graphite, it can be seen that the graphite did not start the pyrolysis process at 500 °C, which reveals that the process of preparing GOs significantly damaged the skeletal structure of graphite, resulting in their poor thermal stability. Correspondingly, two strong endothermic peaks centered at ca. 200 °C and ca. 500 °C were clearly observed in the DSC curves (Figure 3c).

#### 3.1.4. XRD Analysis

The XRD patterns of graphite and GOs showed that the graphite powder presents a very intense and narrow peak at 26.6°, which is attributed to the graphite spacing (0 0 2) of graphite plane (Figure 3d) [48]. After the oxidation of graphite by three different methods, the (0 0 2) peak of graphite disappears and another strong peak appears at about 8°, which is attributed to the typical diffraction peak of GOs [44]. This illustrates the transformation of the crystal structure of graphite into that of GO by the oxidation reaction. The above results indicate that three types of GOs were successfully prepared. In addition, the Bragg equation was used to calculate the lamellar spacing of 0.34 nm, 0.90 nm, 1.37 nm and 0.99 nm for graphite, GO-M1, GO-M2 and GO-M3, respectively. The layer spacing of GOs is significantly larger than that of graphite, which proves that a large number of oxygen-containing functional groups were introduced into the lamellae during the preparation of GOs, resulting in a larger layer spacing. Finally, it is worth noting that the peak GO-M3 at about 8° is the narrowest and highest compared to the peaks of GO-M1 and GO-M2, which can be attributed to the largest crystal grain and the best crystallization of GO-M3.

#### 3.1.5. BET Analysis

**Figure 4:**
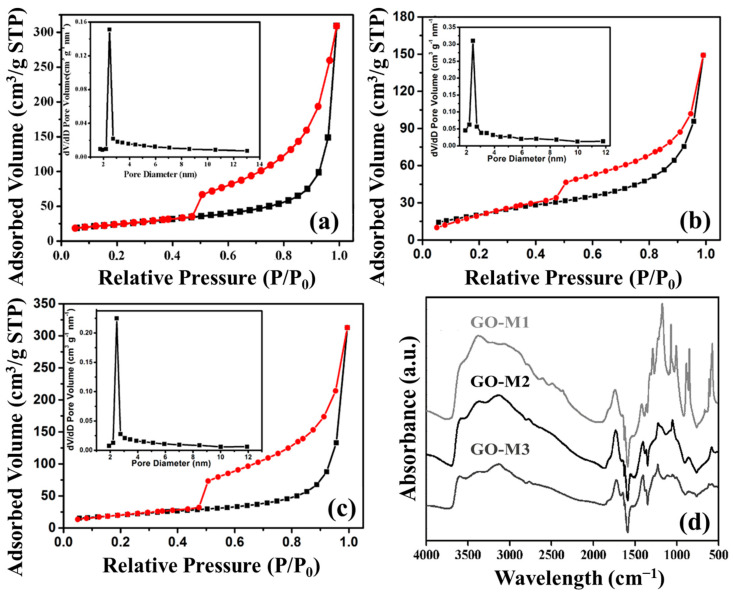
Nitrogen adsorption (black lines) and desorption (red lines) isotherms and pore size distributions of GOs: (**a**) GO-M1, (**b**) GO-M2 and (**c**) GO-M3; FTIR patterns of GOs (**d**).

Nitrogen adsorption and desorption isotherms and pore size distributions of GOs are shown in Figure 4a–c. Judging from the graphs, all three types of GOs are common in mesoporous materials with type IV(a) isotherms, and H3 hysteresis loops that are mostly seen in aggregates of layer-like pore structures and mesoporous materials that produce slit pore structures [49]. Moreover, according to the pore size distribution plots, we discovered that the pore sizes of GO-M1, GO-M2 and GO-M3 were concentrated at 2.45 nm, 2.46 nm and 2.47 nm, respectively. The pore volume of the obtained three materials are 0.0314 cm/g, 0.0204 cm/g and 0.0223 cm/g. Finally, the specific surface areas of the three types of GOs (GO-M1, GO-M2 and GO-M3) were 85.27 m^2^/g, 69.44 m^2^/g, and 88.14 m^2^/g, respectively, according to the measured results of BET characterization showed in Table 3. This implies that the specific surface area of GO-M1 and GO-M3 is much larger than that of GO-M2, which is consistent with the SEM characterization results.

#### 3.1.6. FTIR Analysis

Figure 4d offers the FTIR patterns of GO-M1, GO-M2 and GO-M3. The broad absorption bands at 3000~3700 cm^−1^ are all attributed to -OH/H_2_O, specifically, the peaks ca. 3150 cm^−1^ are the overtones of scissor vibrations of water molecules adsorbed by GOs, the peaks around 3400 cm^−1^ are closely adjacent hydroxyl groups, and the peaks ca. 3600 cm^−1^ are C-OH stretches connected with five-membered-ring lactols and hydroxyl groups of GOs from the basal plane as well as the nanosheet margins [50]. The absorption peaks at ca. 1723 cm^−1^ and ca. 1623 cm^−1^ can be explained by the C=O stretching of the carboxyl and/or carbonyl part of the functional groups [51,52]. The peak at around 1560 cm^−1^ corresponds to the stretching vibration of C=C on the GO skeleton [53]. The 1250 cm^−1^ indicates the C-O stretching of the epoxy group and 1040 cm^−1^ belongs to the C-O-C stretching of the alkoxy group [54]. The above information demonstrates very clearly that the three types of GOs were prepared successfully, and all contain a host of O-containing functional groups, including hydroxyl, carbonyl, carboxyl and epoxy groups.

#### 3.1.7. XPS Analysis

**Figure 5:**
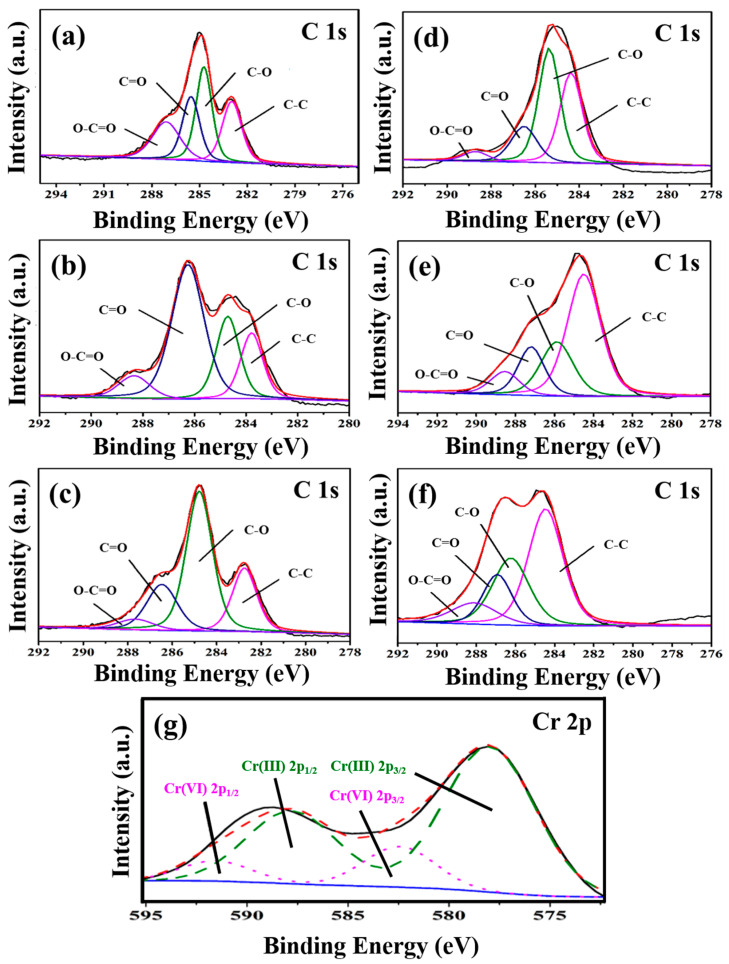
C1s XPS spectra of GO-M1 (**a**), GO-M2 (**b**) and GO-M3 (**c**) before adsorption of Cr(VI); C1s XPS spectra of GO-M1 (**d**), GO-M2 (**e**) and GO-M3 (**f**) after the adsorption of Cr(VI); and the Cr2p XPS spectrum of GO-M3 after the adsorption of Cr(VI) (**g**); the unlabeled blue, black and red lines in the figure are the background, experimental and envelope curves, respectively. Table 4 contains the percentages of each group content to the total groups before and after the adsorption reaction of the three types of GOs calculated based on the results of the split-peak fitting of C 1s. The percentage of C-C groups of the three types of GOs increased significantly after the adsorption reaction (see Table 4), which was mainly due to the reduction of the oxygen-containing groups of the GOs during the adsorption process. It is noteworthy that the C-C group content of GO-M2 rose steeply from 17.4% to 52.4% after the adsorption reaction.

Figure 5 presents the XPS spectra of C 1s of three types of GOs before (Figure 5a–c) and after (Figure 5d–f) the adsorption of Cr(VI), the C 1s spectrum can be divided into four main peaks with the corresponding peak positions and group species as follows: 284.8 eV (C-C), 286.2 eV (C-O), 287.8 eV (C=O) and 289.0 eV (O-C=O) [29]. Figure 5g shows the Cr2p XPS spectrum of GO-M3 after the adsorption of Cr(VI). Typically, the binding energies of Cr 2p_3/2_ at ~578 eV and Cr 2p_1/2_ at ~588 eV refer to Cr(III), while the binding energies of Cr 2p_3/2_ at ~582.5 eV and Cr 2p_1/2_ at ~591.5 eV correspond to Cr(VI). This phenomenon implies that both Cr(VI) and Cr(III) are present on the surface of GO-M3, which is mainly due to the fact that Cr(VI) is immobilized on the surface of GO-M3 by adsorption, and a large portion of Cr(VI) that is anchored on the surface of GO-M3 reacts with a large number of oxygen-containing functional groups on the surface of GO-M3 by redox reaction, thus generating a large amount of Cr(III) [55,56,57].

Here, the group C-O represents two kinds of groups: hydroxyl and epoxy groups, i.e., C-OH and C-O-C. The content of C-O groups before and after the adsorption of GO-M1 increased from 31.1% to 43.1%, which was mainly due to the fact that part of C=O and O-C=O groups were reduced to C-O when GO-M1 adsorbed Cr(VI), thus making the content of C-O groups increase. The content of C-O groups before and after the adsorption of GO-M2 remained basically unchanged, while the content of C-C groups increased significantly, which indicated that the C=O and O-C=O groups were more completely reduced to C-C during the adsorption of Cr(VI) by GO-M2. The content of C-O groups before and after the adsorption reaction of GO-M3 decreased significantly from 53.3% to 27.6%, while the content of C-C groups increased significantly, indicating that some C-O groups were reduced to C-C during the adsorption of Cr(VI) by GO-M3.

The group C=O is mainly the carbonyl group within the lamellae, and the basic structural formula is R-C=O. The content of this group in GO-M1 and GO-M3 decreased slightly before and after the adsorption occurred, while the content of this group in GO-M2 showed a steep decrease. This suggests that the C=O in GO-M1 and GO-M3 hardly participate in the reaction during the adsorption of Cr(VI), while the C=O in GO-M2 plays a major role in the adsorption of Cr(VI).

The group O-C=O is mainly a carboxyl group at the edge of the lamellae. For GO-M2, the content of this group did not appear significant changes before and after adsorption, which means that the carboxyl group in GO-M2 did not participate in the adsorption reaction; for GO-M3, the content of this group in the adsorption reaction showed a slight increase, which reveals that a small portion of other types of oxygen-containing groups was oxidized to carboxyl groups in the adsorption reaction. Remarkably, after the adsorption reaction, the content of this group in GO-M1 decreased from 21.5% to 4.2%, which is a large decrease, indicating that for GO-M1, Cr(VI) mainly reacts with the carboxyl group at the edge of GOs.

According to the above analysis, the carboxyl group on the edge of GO-M1, the carbonyl group on GO-M2 and the hydroxyl and epoxy groups on GO-M3 were mainly involved in the reaction of the three types of GOs during the adsorption of Cr(VI).

### 3.2. Effect of Variables on Adsorption Ability

#### 3.2.1. Effect of Initial Concentration

**Figure 6:**
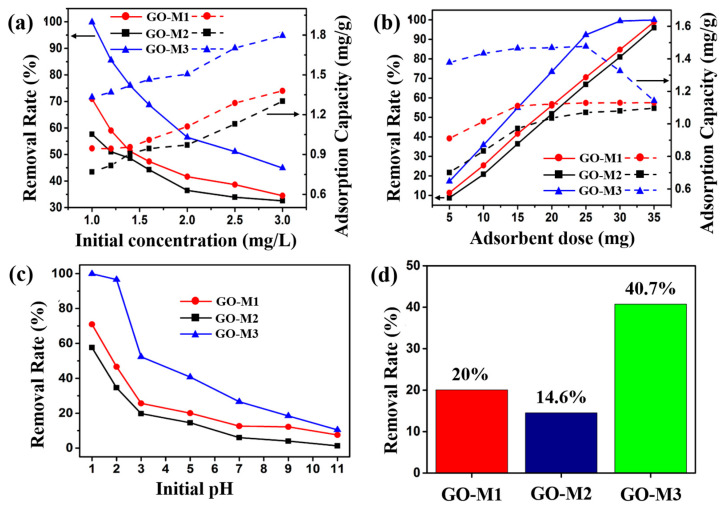
Effect of initial concentration (**a**), adsorbent dose (**b**) and initial pH (**c**) on the adsorption of Cr(VI) by GOs. (**d**) Comparison of the removal of Cr(VI) by GOs at pH = 5 (reaction conditions: the dosage of adsorbent was 15 mg, the initial concentration of Cr(VI) solution was 1 mg/L, the solution volume was 20 mL and the adsorption was carried out at 298.15 K for 30 min).

The effect of the initial concentration of the solution (1.0, 1.2, 1.4, 1.6, 2.0, 2.5, 3.0 mg⋅L^−1^) on the performance of Cr(VI) adsorption by the three types of GOs is illustrated in Figure 6a. Other experimental conditions: adsorbent addition of 15 mg, solution volume of 20 mL, pH of 1, 30 min at 298.15 K. As can be seen in Figure 6a, when the initial solution concentration was 1.0 mg L^−1^, the removal rates of Cr(VI) by GO-M1, GO-M2 and GO-M3 were 70.1%, 57.6% and 99.8%, respectively, and the adsorption capacities were 0.94 mg⋅g^−1^, 0.77 mg⋅g^−1^ and 1.32 mg⋅g^−1^. With the increase of the initial solution concentration, the removal rates of Cr(VI) by GOs decreased gradually, while the adsorption amount increased gradually. When the initial concentration of the solution was 3.0 mg⋅g^−1^, the removal rates of Cr(VI) by GO-M1, GO-M2 and GO-M3 decreased to 34.5%, 32.5% and 44.9% and the adsorption capacities increased to 1.38 mg⋅g^−1^, 1.30 mg⋅g^−1^ and 1.79 mg⋅g^−1^. This phenomenon is due to the continuous adsorption of Cr(VI) by the active adsorption sites on the GOs as the initial concentration of the Cr(VI) solution increases, but with the gradual saturation of the active adsorption sites on the GOs, the saturation adsorption phenomenon eventually appears; with the increase of the initial concentration of the solution, when the Cr(VI) in the solution is larger than the adsorption capacity of the injected GOs, the phenomenon of the reduction of the removal rate occurs [22,58]. It is noteworthy that GO-M3 provides the highest adsorption capacity and removal rate at different initial concentrations when compared to both GO-M1 and GO-M2.

#### 3.2.2. Effect of Adsorbent Dose

The effect of the adsorbent dose (5, 10, 15, 20, 25, 30, 35 mg) on the performance of Cr(VI) adsorption by the three types of GOs is illustrated in Figure 6b. Other experimental conditions: initial solution concentration of 2 mg/L, solution volume of 20 mL, pH of 1, 30 min at 298.15 K. The removal rate and adsorption amount of Cr(VI) by the three adsorbents increased with the increase of adsorbent dosage as seen in Figure 6b, which is attributed to the fact that the reactable adsorption sites increased accordingly with the increase of adsorbent dosage. When the dosage reached 25 mg, the curve of the adsorption amount of GO-M3 decreased, while the removal rate was close to 100%, which was attributed to the fact that almost all the Cr(VI) was removed from the solution, while the adsorbent had not yet reached adsorption saturation [59,60]. Similarly, GO-M3 exhibited the highest adsorption capacity and removal rate at different adsorbent doses compared to GO-M1 and GO-M2.

#### 3.2.3. Effect of Initial pH

The effect of the initial pH (1, 2, 3, 5, 7, 9, 11) on the performance of Cr(VI) adsorption by the three types of GOs is illustrated in Figure 6c. Other experimental conditions: the initial solution concentration of 1 mg/L adsorbent dosage of 15 mg, the solution volume of 20 mL and adsorption at 298.15 K for 30 min. Figure 6c exhibits the removal rate curves of the three types of GOs for Cr(VI) at different pH conditions, from which it can be seen that the removal rate of Cr(VI) showed an obvious decreasing trend with the increase of pH. Specifically, the removal rates of Cr(VI) by GO-M1, GO-M2 and GO-M3 were 80.0%, 57.6% and 99.8%, respectively, at pH 1; while the removal rates were only 7.5%, 1.3% and 10.4%, respectively, at pH 11. Equally, GO-M3 in comparison to GO-M1 and GO-M2 displayed the highest removal rate at different initial pH values.

In general, the effect of pH on the adsorption of heavy metal ions in solution depends on two main aspects: the first is the state of presence of heavy metal ions in solution at different pH conditions; the second is the type of adsorbent surface groups. Moreover, depending on the solution pH and Cr(VI) concentration, Cr(VI) mainly exists in solution in three forms: CrO_4_^2−^ and Cr_2_O_7_^2−^ as well as HCrO_4_^−^ [61]. At 1 < pH < 7, the main form of Cr(VI) in solution is HCrO_4_^−^; at pH > 7, HCrO_4_^-^ in solution is converted to Cr_2_O_7_^2−^; while at pH > 9, Cr(VI) in solution is existing only in the form of CrO_4_^2−^ in solution [62,63]. In addition, low solution pH promotes the redox reaction between the liquid and solid phases due to the large amount of H^+^ in solution participating in the redox reaction of Cr(VI), the specific reaction process of which can be described by Equation (11) [64].

(11) $$HCrO_{4}^{-}+7H^{+}+3e^{-}\to \;Cr^{3+}+4H_{2}O$$

As the pH increases, the predominant form of Cr(VI) present in the solution changes from HCrO_4_^−^ to CrO_4_^2−^. Due to the lower redox potential of CrO_4_^2-^, its oxidation capacity is significantly weaker than that of HCrO_4_^−^. After redox, then the hydrated precipitation of Cr(III) occurs (see Equation (12)) [65].

(12) $$CrO_{4}^{2-}+4H_{2}O+3e^{-}\to Cr{\left(OH\right)}_{3}↓+5OH^{-}$$

Under the same adsorption conditions, the removal rates of Cr(VI) by GO-M1, GO-M2 and GO-M3 under acidic conditions (pH = 5) were 20.0%, 14.6% and 40.7%, respectively, as depicted in Figure 6d, with the removal rates in the order of GO-M3 > GO-M1 > GO-M2. The following four reasons may explain this phenomenon.

### 3.3. Adsorption Kinetics Investigation

**Figure 7:**
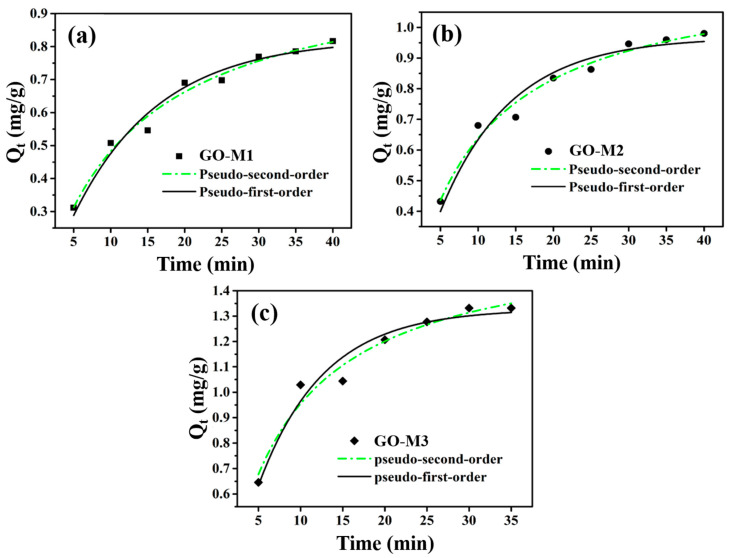
The adsorption kinetics curves of Cr(VI) adsorption by GO-M1 (**a**), GO-M2 (**b**) and GO-M3 (**c**) fitted with nonlinear pseudo-first-order kinetic and nonlinear pseudo-second-order kinetic models (reaction conditions: initial concentration of Cr(VI) 1 mg/L at pH 1, 20 mL of solution, 15 mg of adsorbent, 5~35/40 min, 298.15 K).

The adsorption rate of the adsorbent is one of the core parameters in the adsorption process, as it indicates to some extent the potential of that adsorbent for practical applications. In the study of adsorption processes, adsorption kinetics is the most effective way to characterize the adsorption rate of an adsorbent. Figure 7 demonstrates the adsorption kinetics curves of Cr(VI) adsorption by GO-M1, GO-M2 and GO-M3 fitted using the nonlinear pseudo-first-order kinetic and nonlinear pseudo-second-order kinetic models, respectively. As can be seen in Figure 7, the pseudo-second-order kinetic model describes the kinetic processes of Cr(VI) adsorption by the three types of GOs more closely than the pseudo-first-order kinetic model in the experimental situation; the adsorption of Cr(VI) by all three types of GOs is able to reach the adsorption equilibrium at about 30 min.

The calculation parameters of kinetics for the adsorption of Cr(VI) on GO-M1, GO-M2 and GO-M3 are listed in Table 5, and according to the data in this table, the correlation coefficients (R^2^) of the pseudo-second-order model are all larger than those of the R^2^ of pseudo-first-order model. Therefore, a conclusion can be drawn that the kinetic processes of Cr(VI) adsorption by the three types of GOs conform to the pseudo-second-order kinetic equation, which further implies that these adsorption processes are chemically reaction-controlled [66]. Furthermore, it can be seen from Table 5 that the parameter k_2_ of GO-M3 was 0.0968 g⋅mg^−1^⋅min^−1^ and the parameter Q_e_ of GO-M3 was 1.6187 mg⋅g^−1^, both of which were significantly superior to the other two types GOs (GO-M1 and GO-M2). This suggests that GO-M3 is significantly better than GO-M1 and GO-M2 in terms of adsorption kinetic performance.

### 3.4. Adsorption Isotherms Study

**Figure 8:**
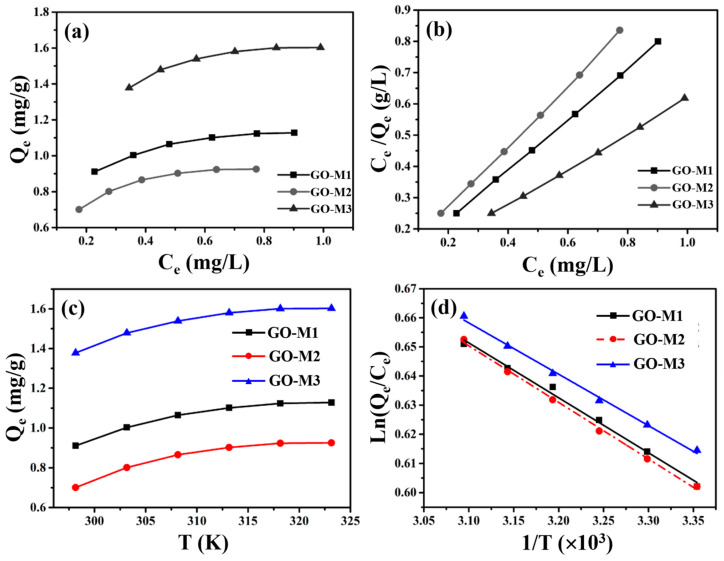
Adsorption isotherms at 298.15 K (**a**) and linear fitting of Langmuir model (**b**) for Cr(VI) adsorption by GOs; the relationship between the equilibrium adsorption capacity of GOs for Cr(VI) removal and temperature (**c**) and the relationship between Ln(Q_e_/C_e_) and 1/T (**d**).

Information on the distribution of Cr(VI) between the liquid and solid phases at equilibrium is provided by the adsorption isotherms. The adsorption isotherms (298.15 K) and linear fitting of Langmuir model for Cr(VI) adsorption by GOs are depicted in Figure 8. As depicted in Figure 8a, GO-M3 possesses the largest equilibrium adsorption capacity, followed by GO-M1 and finally GO-M2.

Subsequently, the linear Langmuir and Freundlich isotherm models were used to fit the data in Figure 8a, and the results are summarized in Table 6 and Figure 8b. We can easily see that the linear Langmuir isotherm model fits the isotherm data in a superior way compared to the linear Freundlich isotherm model, because it provides an R^2^ value very close to 1 [58]. This means that the adsorption process of all three types of GOs on Cr(VI) in solution is consistent with monolayer adsorption [67]. Most importantly, the saturated adsorption capacities (298.15 K) of GO-M1, GO-M2 and GO-M3 for Cr(VI) were predicted to be 3.5412 mg⋅g^−1^, 2.3631 mg⋅g^−1^ and 7.0358 mg⋅g^−1^, respectively, from the above-mentioned adsorption isotherms study.

### 3.5. Adsorption Thermodynamics Study

In order to investigate the spontaneity of the Cr(VI) adsorption process by three types of GOs, adsorption thermodynamic study was carried out. Figure 8c plots the equilibrium adsorption amounts of the three types of GOs for Cr(VI) adsorption versus temperature, from which it can be noticed that the adsorption amounts of the three types of GOs for Cr(VI) increased with the increase of temperature, indicating that the high temperature is favorable for these adsorption reactions and these adsorption processes are endothermic in nature [68]. In addition, we also plotted ln(Qe/Ce) versus 1/T and performed a linear fit to the above data (Figure 8d); the fit was excellent, as these R^2^ were all greater than 0.99.

At the same time, some values of the many calculation parameters of thermodynamics for the adsorption of Cr(VI) on GOs were obtained and are listed in Table 7. As can be seen in Table 7, the Δ*H^θ^* values at 298.15 K to 323.15 K for all three adsorption systems are positive, confirming that these adsorption processes are endothermic processes, which is consistent with the increase in the adsorption amount with increasing temperature [69]. In addition, Δ*S^θ^* values at 298.15 K to 323.15 K are positive for all adsorption systems, indicating an increase in the disorganization of the solid–liquid contact interface during these adsorption processes [70]; Δ*G^θ^* values at 298.15 K to 323.15 K are negative for all adsorption systems studied in this study, confirming that these adsorption processes are thermodynamically spontaneous and feasible [71].

### 3.6. Comparison of GOs with Other Adsorbents for Cr(VI) Adsorption

The Cr(VI) adsorption capacity was usually determined by the Langmuir or Freundlich adsorption isotherms. The previous literature has shown that various materials, such as activated carbon, calcined bauxite, humic acid, zeolite and clay, have been used for the removal of Cr(VI) from aqueous solutions. In this study, the adsorption capacities of different adsorbents were compared with GOs as shown in Table 8. It can be seen from Table 8 that the adsorption capacity of GO-M3 for Cr(VI) was significantly higher than other adsorbents, while GO-M1 and coconut tree sawdust-based activated carbon showed moderate adsorption performance, and GO-M2, calcined bauxite and humic acid showed lower levels of adsorption capacity, while C-HDTMA zeolite and bentonite clay showed the lowest levels of adsorption capacity. The adsorbents of the present study (GOs) showed relatively excellent adsorption capacity, especially CO-M3.

### 3.7. Underlying Adsorption Mechanisms

**Figure 9:**
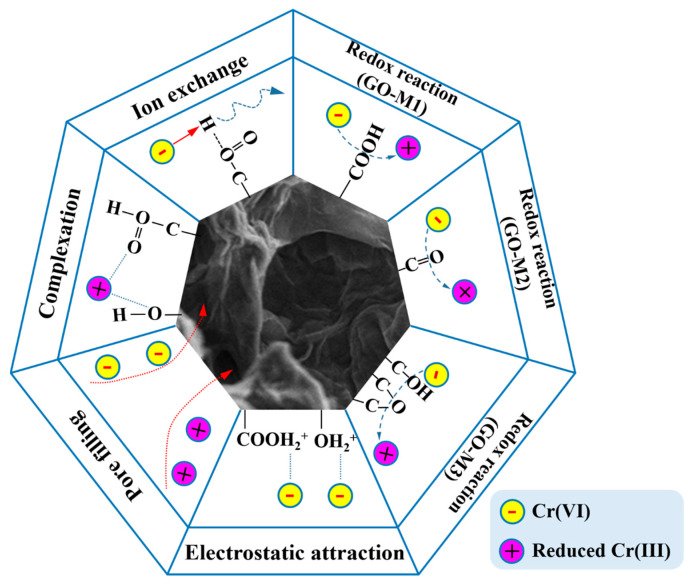
The underlying mechanisms of Cr(VI) adsorption on GOs (different lines represent different forces, and arrows represent the direction of the force).

Figure 9 graphically depicts the underlying mechanisms of Cr(VI) adsorption on GOs. Firstly, numerous O-containing groups, such as hydroxyl and epoxy groups, are contained on GOs. Under acidic conditions, these oxygen-containing groups can combine with H^+^ in solution to form positively charged structures (-OH_2_^+^, -COOH_2_^+^) that subsequently electrostatic interaction electrostatic attraction with the negatively charged Cr(VI) in solution [77,78].

Secondly, under acidic conditions, Cr(VI) in the solution can also undergo redox reactions with oxygen-containing groups [79]. From Table 3, it can be seen that GO-M3 contains the most abundant and chemically active epoxide structure, and this structure allows GO-M3 to most easily react with Cr(VI) by redox reaction and form a reticular complex structure; followed by GO-M1, in which the carboxyl group at the edge is more favorable than the C=O inside the GO-M2 lamellae for its contact with Cr(VI) in solution and thus redox reaction.

Thirdly, according to the BET characterization results, the pore size distributions of the three types of GOs were close to each other, while the specific surface areas varied somewhat. Specifically, GO-M3 possesses the largest specific surface area, followed by GO-M1 and finally GO-M2, and thus their adsorption capacities follow this rule. This suggests that pore-filling is also a very important adsorption mechanism in the three adsorption systems studied [80,81].

Fourthly, with the increase of pH, the oxygen-containing groups on the adsorbent surface exhibited negative charge due to ion exchange, and the repulsive effect with Cr(VI) in solution led to the decrease in the adsorption performance [82,83].

Finally, the adsorption performance of the three adsorbents for Cr(VI) under alkaline conditions is still certain, which is due to the strong surface complexation of oxygen-containing groups on the surface of GOs with Cr(VI), and the adsorption amount of GO-M3 under alkaline conditions is still larger than that of GO-M1 and GO-M2 because the complexation ability of epoxy groups is stronger than that of carboxyl groups and carbonyl groups. Meanwhile, the carboxyl groups exposed at the edges of the GO lamellae are more prone to complexation than the internal C=O, leading to the larger adsorption of GO-M1 than GO-M2.

In conclusion, the underlying mechanisms of Cr(VI) adsorption on GOs primarily consist of redox reaction and electrostatic attraction, in addition to pore filling, ion exchange and surface complexation.

### 3.8. Implications of Results to Water Treatment Practice

The results of this research offer several practical implications for the treatment of Cr(VI)-containing wastewater. Firstly, these GOs were shown to be effective adsorbents for Cr(VI) removal by batch adsorption experiments, and if used as a packing material for column adsorption, they can stably achieve the continuous treatment of Cr(VI)-containing wastewater, which is very suitable for the treatment of real wastewater. Furthermore, the preparation of GOs with super adsorption performance using advanced modification methods can greatly improve the adsorption capacity of the adsorbent and can achieve better treatment effects with the least amount of adsorbent.

## 4. Conclusions

In this paper, three types of GOs (GO-M1, GO-M2 and GO-M3) were prepared by three different methods (M1, M2 and M3) and applied to the removal of Cr(VI) from aqueous solutions. Through advanced characterization techniques, such as SEM-EDS, RS, TG-DSC, XRD, BET, FTIR and XPS, the successful preparation of the three types of GOs was verified on the one hand, and the different mechanisms of Cr(VI) adsorption by the three types of GOs in aqueous solution were revealed on the other hand. Furthermore, various experimental parameters affecting the adsorption of Cr(VI), including the initial concentration and initial pH, adsorbent dosage, contact time and temperature were also investigated. The adsorption kinetics investigation suggested that the pseudo-second-order kinetic model more accurately described the kinetic processes of Cr(VI) adsorption by the three types of GOs and the above adsorption processes were controlled by chemisorption and could reach equilibrium at about 30 min. The adsorption isotherms study indicated that the linear Langmuir model more accurately depicts the adsorption isotherms for Cr(VI) adsorption by the three types of GOs, which suggests that the above adsorption processes are monolayer adsorption; the saturation adsorption capacities (298.15 K) of GO-M1, GO-M2 and GO-M3 for Cr(VI) are expected to be 3.5412 mg⋅g^−1^, 2.3631 mg⋅g^−1^ and 7.0358 mg⋅g^−1^, respectively. The adsorption thermodynamic study showed that these adsorption processes of Cr(VI) by the three types of GOs at 298.15 K to 323.15 K are endothermic, entropy-driven and thermodynamically spontaneous and feasible. More importantly, the redox reaction between Cr(VI) and oxygen-containing functional groups on the surface of GOs is the most essential adsorption mechanism for the adsorption of Cr(VI) by GOs. Specifically, the carboxyl group on the edge of GO-M1, the carbonyl group on GO-M2 and the hydroxyl and epoxy groups on GO-M3 are mainly involved in the reaction of the three types of GOs during the adsorption of Cr(VI). In addition to this adsorption mechanism, electrostatic attraction is also an important adsorption mechanism, and in addition pore filling, ion exchange and complexation are also included. Overall, these experimental results provided vital insights into the mechanism and practical applications of Cr(VI) adsorption by GOs.

## Figures and Tables

**Table 1:** EDS element semi-quantitative analysis results of three types of GOs.

Samples	GO-M1		GO-M2		GO-M3	
Element	Weight (%)	Atom (%)	Weight (%)	Atom (%)	Weight (%)	Atom (%)
C	60.98	68.27	58.79	65.97	55.51	63.23
O	36.53	30.70	39.63	33.38	41.86	35.68
S	2.05	0.86	1.26	0.53	1.71	0.73
Cl	0.43	0.16	0.32	0.12	0.92	0.36
Total	99.99	99.99	100.00	100.00	100.00	100.00

**Table 2:** I_D_/I_G_ values of graphite powder and three types of GOs.

Samples	Graphite	GO-M1	GO-M1	GO-M1
I_D_/I_G_ value	0.113	1.061	1.045	1.069

**Table 3:** BET of graphite powder and three types of GOs.

Samples	GO-M1	GO-M2	GO-M3
The specific surface areas (m^2^/g)	85.27	69.44	88.14
Pore size distribution(nm)	2.45	2.46	2.47
Pore volume(cm^3^/g)	0.0314	0.0204	0.0223

**Table 4:** Chemical states and contents of carbon on the surface of three types of GOs before and after the adsorption of Cr(VI).

Samples	GO-M1		GO-M2		GO-M3	
Chemical States and Contents	Before Sorption	After Sorption	Before Sorption	After Sorption	Before Sorption	After Sorption
C-C	24.5%	36.1%	17.4%	52.4%	22.0%	45.2%
C-O	31.1%	43.1%	22.6%	22.7%	53.3%	27.6%
C=O	22.9%	16.6%	51.6%	16.3%	20.9%	16.3%
O-C=O	21.5%	4.2%	8.4%	8.6%	3.8%	10.9%

**Table 5:** Calculation parameters of kinetics for the adsorption of Cr(VI) on GOs.

Samples	Pseudo-First-Order Model			Pseudo-Second-Order Model		
	*k_1_* (min^−1^)	*Q_e_* (mg⋅g^−1^)	R^2^	*k_2_* (g⋅mg^−1^⋅min^−1^)	*Q_e_* (mg⋅g^−1^)	R^2^
GO-M1	0.1064	0.9679	0.9502	0.0968	1.1913	0.9756
GO-M2	0.0862	0.8236	0.9677	0.0797	1.0560	0.9787
GO-M3	0.1295	1.3285	0.9504	0.0889	1.6187	0.9629

**Table 6:** Calculation parameters of isotherms for the adsorption of Cr(VI) on GOs.

Samples	Linear Langmuir Isotherm Model			Linear Freundlich Isotherm Model		
	*K_L_* (L⋅mg^−1^)	*Q_m_* (mg⋅g^−1^)	R^2^	*K_F_* (mg⋅g^−1^)⋅(L⋅mg^−1^)^1/n^	1/*n*	R^2^
GO-M1	0.3484	3.5412	0.9997	1.4398	0.7112	0.9495
GO-M2	0.4343	2.3631	0.9991	1.5473	0.7589	0.9207
GO-M3	0.2497	7.0358	0.9988	1.3892	0.6775	0.8905

**Table 7:** Calculation parameters of thermodynamics for the adsorption of Cr(VI) on GOs.

Samples	GO-M1			GO-M2			GO-M3		
*T* (K)	Δ*G^θ^* (kJ⋅mol^−1^)	Δ*H^θ^* (kJ⋅mol^−1^)	Δ*S^θ^* (J⋅mol^−1^⋅K^−1^)	Δ*G^θ^* (kJ⋅mol^−1^)	Δ*H^θ^* (kJ⋅mol^−1^)	Δ*S^θ^* (J⋅mol^−1^⋅K^−1^)	Δ*G^θ^* (kJ⋅mol^−1^)	Δ*H^θ^* (kJ⋅mol^−1^)	Δ*S^θ^* (J⋅mol^−1^⋅K^−1^)
298.15	−3.446	3.611	23.67	−3.434	3.715	23.98	−3.502	3.379	23.08
303.15	−3.564			−3.554			−3.617		
308.15	−3.682			−3.674			−3.733		
313.15	−3.801			−3.794			−3.848		
318.15	−3.919			−3.914			−3.963		
323.15	−4.037			−4.034			−4.079		

**Table 8:** A comparison of Cr(VI) adsorption capacity of different adsorbents with GOs.

Adsorbents	Cr(VI) Adsorption Capacity	References
Coconut tree sawdust-based activated carbon	3.46 mg⋅g^−1^	[72]
Calcined bauxite	2.021 mg⋅g^−1^	[73]
Humic acid	2.36 mg⋅g^−1^	[74]
C-HDTMA zeolite	1.6345 mg⋅g^−1^	[75]
Bentonite clay	0.572 mg⋅g^−1^	[76]
GO-M1	3.5412 mg⋅g^−1^	This study
GO-M2	2.3631 mg⋅g^−1^	This study
GO-M3	7.0358 mg⋅g^−1^	This study

## Data Availability

The data is available on reasonable request from the corresponding author.

## Supplementary Materials

The following supporting information can be downloaded at: https://www.mdpi.com/article/10.3390/nano13020279/s1, Text S1: Detailed preparation instructions for three types of graphene oxides.
