# Supplementary material for: Structural Properties of Graphene Oxide Prepared from Graphite by Three Different Methods and the Effect on Removal of Cr(VI) from Aqueous Solution
Source: Nanomaterials (Basel). 2023 Jan 9;13(2):279. doi: 10.3390/nano13020279 (PMC9867312; doi:10.3390/nano13020279)
Supplement: Supplementary file 1 [file nanomaterials-13-00279-s001.zip › nanomaterials-2106181-supplementary.pdf]

nanomaterials

Supplementary Materials

Structural Properties of Graphene Oxide Prepared from Graphite by Three Different Methods and the Effect on Removal of Cr(VI) from Aqueous Solution

Feng Gao [1,2,3], Lei Zhang [1,2], Libin Yang [1,2,3,*], Xuefei Zhou [1,2,3,*] and Yalei Zhang [1,2,3]

[1] State Key Laboratory of Pollution Control and Resources Reuse, College of Environmental Science and Engineering, Tongji University, Shanghai 200092, China
[2] Key Laboratory of Yangtze Water Environment for Ministry of Education, College of Environmental Science and Engineering, Tongji University, Shanghai 200092, China
[3] Shanghai Institute of Pollution Control and Ecological Security, Tongji University, Shanghai, 200092, China
* Correspondence: neuqyanglibin@126.com (L.Y.), zhouxuefei@tongji.edu.cn (X.Z.).

Text S1. Detailed preparation instructions for three types of graphene oxides.

➢ **William S. Hummers Jr.'s method (M1)**

(1) Add 0.5 g graphite powder and 5 g $NaNO_3$ to 100 mL concentrated sulfuric acid, and stir in an ice bath for 1.5 h.

(2) Slowly add 15 g of $KMnO_4$ to the solution of (1) in three times, keep stirring for 1 h, and keep the solution temperature below 5°C.

(3) The solution in (2) was heated to 35°C, condensed and refluxed for 1 h.

(4) Slowly add 120 mL of deionized water to the solution of (3), stir evenly, and then move the solution to an oil bath, raise the temperature to 90°C, and continue stirring for 30 min.

(5) Add 66.5 mL of deionized water and 7 mL of H_2O_2 to the solution of (4) until the solution becomes bright yellow and no more bubbles are formed.

(6) Wash with hydrochloric acid and deionized water with a volume ratio of 1:10 and centrifuge the product to neutrality. After freezing in liquid nitrogen for 5 min, the obtained product is placed in a freeze dryer for 72 h.

➢ **Daniela C. Marcano's method (M2)**

(1) Mix 2 mL of H_2O_2 with 132 mL of deionized water, stir evenly, and place in a refrigerator for 12 h at -40°C.

(2) Mix 120 mL of concentrated sulfuric acid and 13.3 mL of phosphoric acid, add 5 g of graphite powder to the mixed solution, heat up to 50°C, and stir for 12 h.

(3) Pour the solution (2) into (1), the solution turns bright yellow, and let it stand for 12 h.

(4) Wash with hydrochloric acid and deionized water with a volume ratio of 1:10 and centrifuge the product to neutrality. After freezing in liquid nitrogen for 5 min, the obtained product is placed in a freeze dryer for 72 h.

- **Nina I. Kovtyukhova's method (M3)**

Pretreatment stage:

(1) Mix 4.0 g $K_2S_2O_8$, 4.0 g P_2O_5 and 3.0 g graphite powder evenly, slowly add it to 12 mL of concentrated sulfuric acid, heat up to 80°C, and continue stirring for 6 h.

(2) The solution in (1) was cooled at room temperature, diluted with deionized water, and filtered through a filter membrane until neutral, and the obtained filter cake was dried at room temperature for 12 h.

Preparation stage:

(1) Add the pretreated product to 120 mL of concentrated sulfuric acid, and stir in an ice bath.

(2) Slowly add 15 g of $KMnO_4$ to the solution of (1) in three times, keep the solution temperature < 5°C and continue to stir for 30 min, then heat up to 35°C and continue to stir for 2 h.

(3) Add 125 mL of deionized water to the solution of (2) and stir for 15 min.

(4) Add 200 mL of deionized water and 20 mL of H_2O_2 to the solution of (3).

(5) Wash with hydrochloric acid and deionized water with a volume ratio of 1:10 and centrifuge the product to neutrality. After freezing in liquid nitrogen for 5 min, the obtained product is placed in a freeze dryer for 72 h.
